# Antiplatelet Effect of Melatonin through Breastfeeding: A Pediatric Case Report

**DOI:** 10.3390/children10121839

**Published:** 2023-11-23

**Authors:** Sonia Iavarone, Michela Massoud, Giovina Di Felice, Fabio Pulcinelli, Novella Rapini, Matteo Luciani

**Affiliations:** 1 Onco-Hematology, Cell and Gene Therapy and Bone Marrow Transplant Clinic Area, Bambino Gesù Children’s Hospital, IRCCS, 00165 Rome, Italy; sonia.iavarone@uniroma1.it (S.I.); michela.massoud@opbg.net (M.M.); 2 Department of Maternal Infantile and Urological Sciences, Sapienza University of Rome, 00161 Rome, Italy; 3 Clinical Laboratory Unit, Bambino Gesù Children’s Hospital, IRCCS, 00165 Rome, Italy; giovina.difelice@opbg.net; 4 Department of Experimental Medicine, Sapienza University of Rome, 00161 Rome, Italy; fabio.pulcinelli@uniroma1.it; 5 Unit of Endocrinology and Diabetes, Bambino Gesù Children’s Hospital, IRCCS, 00165 Rome, Italy; novella.rapini@opbg.net

**Keywords:** melatonin, breastfeeding, platelets

## Abstract

We present a pediatric case of the antiplatelet effect of melatonin taken through breast milk in an 18-month-old child. The child was referred to our hematology outpatient clinic because of bleeding episodes that she presented since birth. Blood tests excluded the presence of blood coagulation diseases. The family history was negative for bleeding disorders. The child did not consume any drugs, food supplements, herbal teas or infusions. We performed an aggregation platelet test, which showed a reduced platelet aggregation. Shortly before, the baby had been breastfed. We speculated that breast milk could interfere with the result of the test; therefore, we decided to repeat the test in a fasting state. This time the test showed a normal platelet aggregation time. We learned that the child’s mother was taking a mixture of valerian and melatonin. Thus, we decided to suspend maternal intake of melatonin and perform a new platelet aggregation test after three months. The test results were negative. After the suspension of melatonin, the patient did not present further bleeding events. In this case, melatonin, through the inhibition of platelet aggregation, had an important role on the hemostatic system of the child. Melatonin is considered as a dietary supplement and is mostly available as an alternative medicine without formal prescription and dosage regulation. It is important, especially during breastfeeding, to investigate personal and medication history, including also homeopathic remedies or dietary supplements.

## 1. Introduction

Melatonin is a hormone synthesized from tryptophan and secreted by the pineal gland with circadian rhythm [1]. Melatonin’s primary function is its chrono-biotic influence on organismal activity through mediating light and dark signals and thus regulating circadian and seasonal endocrine and non-endocrine rhythms [2]. For this reason, melatonin is widely used for sleep disorders at all ages but especially associated with the menopause or psychiatric diseases or to improve jet lag symptoms [3]. However, several studies showed that melatonin supplementation induces antioxidative, anti-inflammatory, anxiolytic, analgesic, anti-depressive, sedative and immune-modulatory effects, which reclassifies melatonin as a multi-tasking molecule and not only as a hormone [4,5]. The finding of melatonin binding sites in many cells, including the retina, blood lymphocytes, monocytes and platelets [6,7,8], may explain numerous effects and functions of this molecule. Particularly, some studies demonstrated its direct interaction with platelets and showed that melatonin exogenous administration may influence coagulation. Cardinali et al. have shown that the inhibitory effect of melatonin on platelets could be dose-dependent: melatonin stimulates platelet aggregation at physiological levels, while at a medium concentration (>1 µM but <10 µM), it inhibits it, and at higher concentrations, it inhibits platelet cyclooxygenase and reduces arachidonic acid-induced aggregation and thromboxane B2 production [9]. Finally, Girish et al. highlighted that melatonin at a therapeutic dosage has a platelet proapoptotic effect [7].

Based on these data and our recent clinical experience, we report a pediatric case of the antiplatelet effect of melatonin in a child nursed by a mother, who took melatonin throughout pregnancy and during breastfeeding.

## 2. Case Report

We describe a pediatric case of abnormal bleeding in an 18-month-old female child breastfed since her birth. The child was born at term by spontaneous delivery and no complications were reported during pregnancy.

The child was referred to our hematology outpatient clinic because, 3 months earlier, she had presented with prolonged and abnormal bleeding from a small wound caused by accidental trauma to her upper lip. The bleeding had required access to the pediatric emergency department, where blood tests had been performed with coagulation tests found to be normal. The bleeding had stopped partially after the use of tranexamic acid and desmopressin. The child had not taken any medications, dietary supplements, herbal teas or infusions, as reported by the parents. The family history was negative for bleeding disorders. Moreover, the child had presented with intermittent bleeding for four months after the fall of the umbilical cord, which was unsuccessfully treated with local hemostatic procedures. Afterwards, the child did not present other hemorrhagic events, even after an accidental clavicle fracture of unclear etiology. Due to the abnormal bleeding events in the absence of hemostatic disorders, as revealed by an initial hematologic evaluation, and the two previous apparently accidental traumas, the family pediatrician thought of the possibility of Munchausen syndrome.

The child’s mother decided to take her to our Hemostasis Center for a second opinion. Blood tests were performed and showed normal values of blood count, activated partial thromboplastin time (aPTT), prothrombin time (PT), fibrinogen levels, coagulation factors (II, V, VII, VIII, IX, X, XI, XII, XIII) and vWF Antigen (vWF:Ag). The Platelet Function Analyzer 100 (PFA-100) results were also normal. Coagulation disorders were therefore excluded. We decided to perform a platelet aggregation test, that showed reduced platelet aggregation. Upon further investigation of the medical history, it was found that the child had taken breast milk shortly before the test. We speculated that breast milk might have interfered with the result, and we decided to repeat the test in a fasting state. The repeated test showed a normal aggregation time. We then thoroughly examined the mother’s medical history, both personal and pharmacological, investigating her intake of infusions, herbal teas or homeopathic products, and we found that she had taken a mixture of valerian and melatonin both during pregnancy and for a prolonged period during breastfeeding. In particular, the child’s mother had taken a supplement containing a mixture of valerian and melatonin, at a dosage of 1 mg melatonin and 45 mg valerian extract per tablet; she usually took the supplement in the evening, before bedtime, but her medical history showed that she occasionally took melatonin for afternoon rest as well. The mother referred that she did not take more than 10 tablets per day, that is a maximum of 10 mg of melatonin daily.

Subsequently, the mother discontinued the melatonin for several months, and during this period, her daughter no longer presented with bleeding, even during major traumatic events (fracture of the clavicle). For several weeks, the mother resumed taking melatonin and valerian for sleep disorders.

A new platelet aggregation test was performed three months after the mother’s withdrawal of exogenous melatonin. The test result was negative, suggesting that the altered platelet aggregation of the previous test could be caused by the antiplatelet effect of melatonin, administered to the child through breast milk. Since then, the child has not presented any other bleeding episodes.

## 3. Discussion

We reported a pediatric case of the antiplatelet effect of melatonin taken through breast milk in a child, who had experienced abnormal bleeding in the absence of a known cause. What emerged from the clinical history is that the mother had taken a mixture of valerian and melatonin throughout her pregnancy and for a long period during breastfeeding, thinking that, as a supplement, melatonin could not have undesirable effects on the child. Melatonin is a small lipophilic neuro-hormone secreted into the blood by the pineal gland. Its production is suppressed by light and restored at night, which explains why melatonin is involved in the synchronization of the human circadian rhythm [10].

During pregnancy, melatonin plays an important role in prenatal development. Fetal circadian rhythms are closely related to the maternal rhythmic secretion of melatonin and cortisol, and in particular, circadian fluctuations in melatonin levels peak before birth [11]. In addition, melatonin production in the placenta increases, and this feto-maternal connection guides the development of the fetal circadian system until birth. Melatonin levels increase markedly in the last trimester of pregnancy, starting from the 32nd week of gestation, and it is during this period that the circadian rhythm of the fetal heart rate is evident, as well as different types of sleep [12]. After birth, the absence of melatonin physiologically produced by the placenta can be compensated for by its presence in breast milk [13]. After 9–12 weeks of age, physiological circadian changes in the sleep–wake rhythm and body temperature are established [14]. Assuming that the rhythmic production of endogenous melatonin is achieved only from the age of 6 months, it is important to take melatonin through breast milk in the first months of life [15]. Breast milk is the only external source of melatonin; this is especially important in preterm infants, who have an increased risk of developing future cardiovascular disorders [16]. Indeed, preterm infants lack the exposure to higher levels of melatonin that occur in the latter part of pregnancy; they also have a delay in the rhythmic production of endogenous melatonin compared with term infants. However, melatonin in the milk of mothers of preterm infants reaches a higher concentration at peak [17], making up for the deficiency secondary to preterm birth.

Abnormalities in serotonin and melatonin levels in biological fluids have been found in infants with sudden infant death syndrome (SIDS) [18,19]. In contrast to adults, where melatonin has a short half-life of ∼40 min, in preterm infants, melatonin has a half-life of 17–21 h. This makes it difficult to establish a circadian rhythm with the use of exogenous melatonin in infants [20,21]. Another area of concern is the infant’s ability to metabolize xenobiotics: CYP1A2 levels are 20–25% of adult levels in the first year of life and 50% in older children [21]. Therefore, a longer half-life and reduced metabolization could enhance the effects of melatonin in infants.

Endogenous melatonin has been found in mother’s milk 3–4 days after delivery [22,23]. In the milk of lactating mothers, melatonin has a daily circadian peak of ~24 pg/mL, while levels will be undetectable in the remaining part of the day [24]. Circadian fluctuation of melatonin in milk might influence the circadian rhythm of breastfed infants [25]. To date, there are no studies on the levels achieved by melatonin in breast milk after exogenous supplementation, as the scientific community advises against taking melatonin in pregnancy and lactation [26]. Bishop-Freeman et al., reported seven cases of undetermined pediatric deaths, in which high melatonin levels were detected during a postmortem blood toxicology analysis, consistent with exogenous supplementation. No data are available on the acute toxicity of melatonin, but it may cause numerous systemic effects through mechanisms of action that are not fully elucidated [27]. Oral melatonin undergoes first-pass metabolism by hepatic cytochrome P450 enzymes, mainly CYP1A2. DeMuro et al., in their study, showed that 85% of melatonin, administered at an oral dose of 2 mg and 4 mg, undergoes first-pass metabolism, while only 15% of the ingested dose reaches the systemic circulation [28]. During pregnancy and breastfeeding, the maternal body undergoes physiological changes that affect drug metabolism, pharmacokinetics and drug bioavailability. Therefore, we could hypothesize that a continuous and prolonged intake of exogenous melatonin during pregnancy and lactation, coupled with changes in melatonin metabolism during this period, could be responsible for the increased bioavailability of melatonin in breast milk.

Melatonin binding sites have been identified on several cells, including platelets [6,8]. Several studies have demonstrated its direct interaction with platelets and have shown that the exogenous administration of melatonin can affect the hemostatic system [9,29]. Melatonin appears to exert a dose-dependent inhibition on platelet activity. Vacas et al. not only observed high-affinity binding sites for [3H]-melatonin on the platelet membrane, but also described a model of inverse sensitivity of platelets to melatonin [29].

Specifically, melatonin would appear to inhibit platelet aggregation, adenosine triphosphate (ATP) and serotonin release; it also inhibits thromboxane B2 production. Based on this assumption, melatonin could play a therapeutic role in coronary disease by inhibiting these processes in platelets [30]. In infants taking melatonin through breast milk, there are positive effects on the establishment of cardiovascular system rhythms, which are essential for future cardiovascular health [31]. Cardinali et al. showed the presence of melatonin binding sites on platelets and a dose-dependent inhibitory effect on platelet aggregation, inhibiting the production of arachidonic acid (AA) and adenosine diphosphate (ADP) in platelet-rich plasma (PRP) [9,32]. According to Kornblihtt et al., the marked inhibitory effect of melatonin is due to its action on collagen, AA, ADP, epinephrine and inhibition of collagen-induced platelet activation. In addition, melatonin acts at an earlier stage in the cyclooxygenase-dependent pathway [33]. Bohm et al. demonstrated that melatonin reduces thrombin-induced platelet aggregation in vitro, especially in healthy subjects [34]. In addition, platelets appear to exhibit circadian responsiveness to melatonin, with the antiplatelet-aggregating effect of melatonin on platelets being greater at night [35]. Girish et al. have shown that melatonin administration results in increased platelet apoptosis, probably secondary to increased intracellular calcium and reactive oxygen species (ROS) levels [7]. These effects could result in thrombocytopenia. In a study of patients with hemorrhagic stroke, it was observed that melatonin administration at a dose of 30 mg/d for 5 days resulted in decreased levels of fibrinogen, VII and Von Willebrand factors (VWFs) and prolonged the prothrombin time [36]. The development of hemostasis begins in utero, evolving gradually during gestation with major changes beginning in the first weeks of life. Beginning at birth, there is a gradual increase in the concentrations of coagulation factors. In particular, vitamin K-dependent coagulation factors (II, VII, IX, X) in infants have concentrations at 50% of adult values [37]; in addition, the function of platelets and fibrinogen and the ability to generate thrombin are also reduced [38,39].

Bleeding time provides information about the vaso-platelet phase of hemostasis and is shorter in healthy infants than in adults, probably due to high hematocrit values, the presence of large red blood cells and higher concentration of high molecular weight von Willebrand factor (VWF) multimers [40]. It is speculated that in the newborn, the combination of lower physiological levels of coagulation factors and the altered platelet function may interfere with the physiological hemostatic process by predisposing to hemorrhage; in addition, exposure of the breastfed infant to substances that may interfere with primary and secondary hemostasis could increase the risk of hemorrhage. In infants, a decrease in plasma concentrations of factors VII, X, V, II or fibrinogen results in prolonged PT, while a decrease in plasma levels of FXII, FXI, FVIII and FIX is associated with prolonged APTT. In infants FVIII, FV, and FXIII levels are similar to those in adults. Although plasma concentrations of fibrinogen in the newborn are higher, due to the presence of “fetal” fibrinogen, which exhibits delayed polymerization, the thrombin clotting time is prolonged in newborns [41]. In the Wirtz et al. study, a decrease in plasma concentrations of fibrinogen, factor VIII and D-dimer in healthy young men was observed one hour after administration of 3 mg melatonin [42]. In another study, a reduction in coagulating activity was observed, with lower levels of fibrinogen and FVIII, following exposure to higher doses of melatonin [43].

Based on this evidence, we can hypothesize that in our clinical case, the bleeding in the first few months of life, following umbilical cord fall, was probably due to both the antiplatelet effect of melatonin and the interference of melatonin itself with secondary hemostasis, considering that in the first weeks of life, the levels of coagulation factors are already physiologically reduced. When the child came to our observation, we found no abnormalities in platelet, aPTT, PT, fibrinogen and coagulation factor counts; this was likely due to the progressive and physiological increase in fibrinogen and coagulation factor levels during growth. However, our laboratory tests slated the antiplatelet effect of melatonin through a platelet aggregation test.

Several studies have shown that melatonin exhibits very low toxicity when administered at various doses [44]. In particular, for every 1 mg of exogenous melatonin consumed by the mother, an increase from 0.4 to 1 mcg/L of its concentrations in breast milk is observed. However, a prolonged intake of melatonin for many months could alter not only the development of the circadian rhythm in children, but also interfere with the hemostatic system [25]. However, there are no sufficient data in the literature concerning exposure to exogenous melatonin in pregnant women. Although melatonin’s toxic effects have not been found in in vivo studies on pregnancy, embryo–fetal development, childbirth and postpartum, melatonin supplementation during pregnancy and breastfeeding is not recommended due to the lack of studies on melatonin interaction with maternal milk [26]. To date, there are no studies that allow us to establish the safety of using supplements during pregnancy and breastfeeding [45]. Therefore, healthcare professionals should inform mothers about the possible risks of taking supplements both during pregnancy and lactation.

## 4. Conclusions

To the best of our knowledge, we described the first case of melatonin’s antiplatelet effect, taken through breast milk, in a female child aged 18 months. Through our case, we have highlighted the importance of investigating the mother’s medical history, both personal and pharmacological, relating to the intake of supplements or homeopathic therapy. Melatonin is usually taken as a supplement and can be purchased without a prescription; however, it is important that healthcare professionals provide information to mothers about the use of supplements, safety profiles and the risk–benefit ratio during lactation. This case report highlights that melatonin should be taken with caution during lactation and should be discontinued after a history of infant bleeding. However, the underlying pathophysiological mechanisms are still unclear and further research is needed to provide recommendations and implement our current knowledge.

## Data Availability

Data are contained within the article.
